# OrthoML2GO: homology-based protein function prediction using orthogroups and machine learning

**DOI:** 10.18699/vjgb-25-119

**Published:** 2025-12

**Authors:** E.V. Malyugin, D.A. Afonnikov

**Affiliations:** Novosibirsk State University, Novosibirsk, Russia; Institute of Cytology and Genetics of the Siberian Branch of the Russian Academy of Sciences, Novosibirsk, Russia

**Keywords:** protein function prediction, gene ontology, homology, orthogroup, machine learning, предсказание функций белка, генная онтология, гомология, ортогруппа, машинное обучение

## Abstract

In recent years, the rapid growth of sequencing data has exacerbated the problem of functional annotation of protein sequences, as traditional homology-based methods face limitations when working with distant homologs, making it difficult to accurately determine protein functions. This paper introduces the OrthoML2GO method for protein function prediction, which integrates homology searches using the USEARCH algorithm, orthogroup analysis based on OrthoDB version 12.0, and a machine learning algorithm (gradient boosting). A key feature of our approach is the use of orthogroup information to account for the evolutionary and functional similarity of proteins and the application of machine learning to refine the assigned GO terms for the target sequence. To select the optimal algorithm for protein annotation, the following approaches were applied sequentially: the k-nearest neighbors (KNN) method; a method based on the annotation of the orthogroup most represented in the k-nearest homologs (OG); a method of verifying the GO terms identified in the previous stage using machine learning algorithms. A comparison of the prediction accuracy of GO terms using the OrthoML2GO method with the Blast2GO and PANNZER2 annotation programs was performed on sequence samples from both individual organisms (humans, Arabidopsis) and a combined sample represented by different taxa. Our results demonstrate that the proposed method is comparable to, and by some evaluation metrics outperforms, these existing methods in terms of the quality of protein function prediction, especially on large and heterogeneous samples of organisms. The greatest performance improvement is achieved by combining information about the closest homologs and orthogroups with verification of terms using machine learning methods. Our approach demonstrates high performance for large-scale automatic protein annotation, and prospects for further development include optimizing machine learning model parameters for specific biological tasks and integrating additional sources of structural and functional information, which will further improve the method’s accuracy and versatility. In addition, the introduction of new bioinformatics tools and the expansion of the annotated protein database will contribute to the further improvement of the proposed approach.

## Abstract (ru)

В последние годы быстрый рост объемов данных секвенирования обострил проблему функциональной аннотации белковых последовательностей, поскольку традиционные методы, основанные на гомологии, сталкиваются с ограничениями при работе с отдаленными гомологами, что затрудняет наиболее точное определение функций белков. В нашей работе представлен метод предсказания функций белков OrthoML2GO, который интегрирует поиск гомологичных последовательностей с помощью алгоритма USEARCH, анализ ортогрупп на базе OrthoDB 12-й версии и алгоритм машинного обучения (градиентный бустинг). Ключевая особенность подхода заключается в использовании информации об ортогруппах для учета эволюционного и функционального сходства белков и применения машинного обучения для дальнейшего уточнения терминов Gene Ontology (GO) для анализируемой последовательности. Для выбора оптимального алгоритма аннотации белков были поэтапно применены следующие подходы: метод k ближайших соседей (KNN); метод на основе аннотации ортогруппы, наиболее представленной у k ближайших гомологов (OG); метод верификации выявленных на предыдущем этапе терминов GO с помощью алгоритмов машинного обучения. Проведено сравнение точности предсказания терминов GO методом OrthoML2GO с программами аннотации Blast2GO и PANNZER2 на выборках последовательностей как отдельных организмов (человек, арабидопсис), так и на комбинированной выборке последовательностей, представленных разными таксонами. Результаты показали, что предложенный метод не уступает, а по некоторым показателям превосходит их по качеству предсказания функций белков, особенно на больших и разнородных выборках организмов, а наибольший прирост точности достигается за счет комбинации информации о ближайших гомологах и ортогруппах в сочетании с верификацией терминов методами машинного обучения. Разработанный подход демонстрирует высокую эффективность для крупномасштабной автоматической аннотации белков. Перспективы дальнейшего развития включают оптимизацию параметров моделей машинного обучения под конкретные биологические задачи и интеграцию дополнительных источников структурно-функциональной информации, что позволит еще больше повысить точность и универсальность метода. Кроме того, внедрение новых инструментов биоинформатики и расширение базы данных аннотированных белков будут способствовать дальнейшему совершенствованию предложенного подхода.

## Introduction

The introduction of next-generation sequencing (NGS) technologies has led to exponential growth in the volume of data on DNA, RNA, and protein sequences (Goodwin et al., 2016). The primary sources of these data are large-scale and numerous projects in genomics, transcriptomics, and proteomics (Cheng et al., 2018; Lewin et al., 2018). However, the function of a significant proportion of the sequences identified in such projects remains unknown (Galperin, Koonin, 2010).

Expert gene annotation requires substantial time to search for gene function information in the literature, and although it is the most reliable method, it is impractical to apply it to the vast number of newly predicted genes. Therefore, for most new amino acid sequences (hereafter referred to as sequences for brevity), the development of effective automatic annotation methods is necessary to determine their molecular functions, roles in cellular processes, and cellular localization. Given the widespread use of the Gene Ontology (GO) database for functional annotation (Ashburner et al., 2000; Du Plessis et al., 2011; Gene Ontology Consortium, 2023), the task reduces to automatically assigning these terms to sequences

Most methods for predicting protein function, based on sequence or three-dimensional structure analysis, rely on a fundamental principle: function can be predicted by establishing reliable structural or evolutionary similarity with a protein, the function of which is already known (Benso et al., 2013). A crucial task here is deciphering the relationship between the detected structural or sequence similarity and the actual level of functional relatedness (Pearson, 2013). Among these methods, homology-based function prediction methods are widely regarded for their broad applicability and relative simplicity. Homology-based methods assign GO terms to the analyzed protein based on the similarity of its amino acid sequence to the primary structures of proteins with known functions. In other words, the function of a protein can be deciphered by analyzing its similarity to other proteins for which the function has been reliably determined (Eisenberg et al., 2000; Pearson, 2013).

The BLAST method (Altschul et al., 1990) is widely used for comparing the amino acid sequences and identifying homologous regions. However, new tools for searching homologous sequences in databases have recently emerged, such as GHOSTX (Suzuki et al., 2014), DIAMOND (Buchfink et al., 2015), MMseqs2 (Steinegger, Söding, 2017), and others. Their characteristic feature is high processing speed, orders of magnitude faster than BLAST, achieved primarily through more efficient processing of matched sequence fragments.

The concept of homology is fundamental for drawing conclusions about the evolutionary processes of gene formation and function. In the early 1970s, Walter Fitch (Fitch, 1970) proposed classifying homologous proteins into orthologs and paralogs according to their origin. Orthologs originate from the evolutionary divergence of genes in different taxa during speciation. Paralogs are formed through gene duplications. It is assumed that orthologs retain the function of the ancestral gene from the ancestral species, while paralogs may acquire new functions after duplication events (Fitch, 2000; Kuzniar et al., 2008; Altenhoff et al., 2019). Given the immense importance of orthologs for comparative genomics and functional annotation, information on orthologous genes and their families is accumulated in several specialized databases, which are crucial for identifying and analyzing orthologous groups of genes (orthogroups) (Jensen et al., 2008; Kriventseva et al., 2008). It should be noted that methods involving machine learning algorithms are successfully used to solve gene function prediction problems, allowing for increased accuracy compared to earlier approaches (Sanderson et al., 2023; Yuan et al., 2023).

This work investigates the possibility of predicting protein functions based on searching for homologous sequences, considering their orthologs, and employing machine learning methods. A step-by-step analysis of the influence of these three factors on the accuracy of GO term prediction was performed. It is shown that among machine learning methods, the gradient boosting algorithm demonstrates the highest prediction accuracy. Based on this, the OrthoML2GO prediction algorithm was implemented. Its accuracy was compared with the Blast2GO and PANNZER2 methods. It is shown that the proposed method provides higher accuracy, especially on large and heterogeneous datasets

## Materials and methods

Amino acid sequence data. The lists of organism species and amino acid sequences used in the work are presented in Table 1. They include organisms with varying degrees of genome annotation completeness (Table S1)1, representing different taxa of both plants and animals: dicots, monocots, unicellular algae, vertebrates, arthropods (Table 1).


Supplementary Materials are available in the online version of the paper: https://vavilov.elpub.ru/jour/manager/files/Suppl_Malugin_Engl_29_7.pdf


OrthoDB as a source of homologous sequences, annotations, and orthology information. The OrthoDB v 12.0 database (https://www.orthodb.org/) (Tegenfeldt et al., 2025) was used as a source of homologous sequences, their GO term annotations, and orthology data. The database includes information on 5,827 eukaryotic species, 17,551 bacteria, 607 archaea, and 7,962 viruses. It contains over 162 million sequences classified into over 10 million orthogroups. The database also includes GO annotation for part of the sequences and thus represents a convenient source for their classification into orthologs and GO annotation. Furthermore, this database provides classification of protein sequences into orthologous families, for which generalized functional annotations of proteins in GO terms are also provided.

Search for homologous sequences. The search for homologs was performed using the USEARCH v 11.0.667 algorithm (https://drive5.com/usearch/) (Edgar, 2010) with the usearch_local command. It performs searches for high-identity matches orders of magnitude faster than BLAST. During the search for homologous sequences, it was inevitable that the list of homologs included the query sequence itself. For an objective evaluation, identical sequences were excluded from the search results.

General sequence annotation scheme. The GO term annotation pipeline was implemented using Linux bash scripts and the R programming language using the computational resources of the “Bioinformatics” collective use center at ICG SB RAS. Three algorithms for annotating protein functions based on the OrthoDB database were developed (Fig. 1).

On the left (Fig. 1a), the OrthoDB v 12.0 database (Tegenfeldt et al., 2025) is schematically shown in a large oval with representatives of orthologous groups (orthogroups) OG1... OG3 (Sequences of orthologous families are shown as rectangles of the same color). The first, basic sequence prediction algorithm is based on the search for k-nearest homologs and is denoted as KNN. Using the USEARCH program, homologous sequences are searched for the analyzed sequence in the OrthoDB database and ranked by similarity level. They can include representatives of both the same orthogroup and others (shown in different colors). The analyzed sequence is assigned the GO terms of the k most similar sequences (Fig. 1b)

The second method is based on the principle of orthology and is denoted as OG. For each of the k-nearest homologs of the analyzed sequence, its orthogroup in the OrthoDB database is determined. The orthogroup to which the analyzed sequence belongs is determined by a voting method: it is the orthogroup with the highest frequency of occurrence among all k-nearest homologs (Fig. 1c). GO terms for sequences from this orthogroup are assigned to the analyzed sequence (Fig. 1d).

The third approach, denoted as KNN+OG (Fig. 1e), involves combining the GO terms obtained from the KNN and OG algorithms for the query sequence (Fig. 1f ). This list of GO terms is compared with the reference (true) annotation using measures such as: precision, recall (sensitivity), accuracy, and F-score (F-measure), which was the resulting measure (Fig. 1g and “Verification of terms using machine learning methods” section).

Methods for annotating the analyzed sequence with GO terms. K-nearest homologs method (KNN ). The k-nearest homologs by similarity level are determined as a result of searching the OrthoDB database with the USEARCH program with the following parameters: identity (amino acid sequence identity) = 50 %, coverage (coverage of the analyzed sequence by the found homolog) = 70 %, e-value (statistical significance of the found match) = 10–6, which is justified by the goal of reducing false positives at the homolog search stage. The analyzed sequence was assigned the GO terms of the k most similar sequences available in the OrthoDB database. The value of parameter k can vary (Kharsikar et al., 2007; Dongardive, Abraham, 2016). Therefore, the optimal value within the interval k = 1–30 with a step of 5 was determined based on the highest accuracy in term identification using the OrthoDB annotation (Tables S4–S9).

Using orthologous groups (OG). In this method, for each of the k-nearest homologs identified by the KNN method, the orthologous group corresponding to the most ancient ancestral taxon was selected using the OrthoDB annotation. Then, the orthogroup with the highest frequency among the k-nearest homologs was determined and assigned to the analyzed sequence. GO annotation terms for sequences from this orthogroup in the OrthoDB database were assigned to the analyzed sequence. The KNN+OG method combines GO terms (excluding duplicates) obtained separately by the KNN and OG methods described above.

Verification of terms using machine learning methods. To refine the list of predicted GO terms at the third stage of analysis (Fig. 1f ), three machine learning (ML) algorithms were employed: logistic regression (LR), gradient boosting (XGB), and random forest (RF). Note that this stage does not allow adding new terms to the annotation. Instead, it filters out terms for which the similarity parameters between the analyzed sequence and its homologs do not meet the specified criteria.

The logistic regression method (LR) is implemented in the built-in stats package (R Core Team, 2013) via the function glm (family = binomial). Logistic regression predicts the probability of an object belonging to a class (e. g., “spam” or “not spam”). It predicts the probability of an object belonging to a class based on a weighted sum of features and passes it through a logistic (sigmoid) function, which normalizes the result to a number (probability) between 0 and 1. Gradient boosting (XGB – eXtreme Gradient Boosting) was used in the variant implemented in the xgboost package (Chen, Guestrin, 2016), function xgb.train. The random forest method (RF) was applied in the version from the randomForest package (Liaw, Wiener, 2002), function randomForest. Both gradient boosting and random forest are ensemble algorithms based on decision trees. This means that the final prediction is the result of the collective work of many individual decision trees. The parameters of the gradient boosting and random forest algorithms are specified in the Table S12.

Parameters for the models were selected during training, and in each method, their set was the same for all GO terms, analyzed sequences, and their homologs. These are terms reflecting the level of similarity, amino acid composition, and frequency of GO terms (Table S2). If a GO term in a homolog was present in the annotation of the analyzed sequence in the training set, the prediction function value in the machine learning method was 1, otherwise, 0.

To evaluate the accuracy of machine learning methods, amino acid sequences of Arabidopsis thaliana and Homo sapiens proteins were used (Table 1). The set of sequences for each of these two species was divided into two parts: 80 % for training and 20 % for testing. Additionally, a combined sample of proteins from the organisms listed in Table 1 was formed: from the combined sample, 50,000 sequences were randomly selected for training, and 20,000 non-overlapping sequences were selected for testing the machine learning models (Table S3).

Evaluation metrics. Annotation accuracy evaluation was performed in R using the dplyr package (Wickham et al., 2025). For this, two lists were formed: (a) a reference list with amino acid sequences annotated with GO terms from databases for model organisms (more details in Table S1) and (b) a list obtained through functional annotation using various annotation methods (Fig. 1). To assess the accuracy of the annotation obtained by each of the methods described above, they were compared with the reference annotation. Hereafter, True Positive (TP) refers to GO terms present in both lists; False Positive (FP) refers to terms present in the predicted annotation list but absent in the reference (true) list; False Negative (FN) refers to terms present in the reference list but absent in the predicted annotation list.

The following metrics were used to evaluate protein annotation: Precision (PR), Recall (RC), Accuracy (AC), as well as the F-score metric, which was the resulting measure (Note. Here, “Accuracy (AC)” is a defined metric, distinct from the general concept of prediction accuracy):

**Formula. 1.**
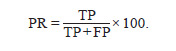
Formula 1

Recall (RC) – the proportion of true positive predictions among all true terms in the reference annotation:

**Formula. 2.**
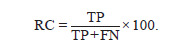
Formula 2

Accuracy (AC) is defined as the arithmetic mean of Precision and Recall

**Formula. 3.**
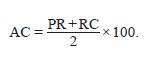
Formula 3

F-score (F-measure) represents the harmonic mean between Precision and Recall. This metric approaches zero if either Precision or Recall approaches zero:

**Formula. 4.**
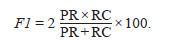
Formula 4

Since machine learning algorithms (LR, XGB, RF) estimate the probability of a GO term belonging to the analyzed sequence, and not a binary decision, it is necessary to choose a cutoff threshold (t) above which the term will be considered predicted. To account for data imbalance and to choose an optimal threshold independent of its specific value, the Fmax metric was calculated for the cutoff threshold t ∈ (0; 1) with a step of 0.1. A GO term was considered correctly predicted (positive class) if its predicted probability exceeded threshold t. Fmax is defined as the maximum value of F-score(t) across all thresholds

**Formula. 5.**
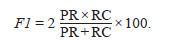
Formula 5

In GO term prediction tasks, where the distribution of terms by frequency of occurrence is extremely imbalanced (some terms are very common, others are extremely rare), and classification is multi-label (one protein can correspond to many terms), the Fmax metric is often used. It is calculated for the entire set of predictions by varying the cutoff threshold (t), above which a term predicted by the ML model is considered positive. Fmax shows the maximum quality that the model can achieve in the ideal case of threshold selection. Unlike the F1- score, which is calculated for a fixed threshold, Fmax evaluates the quality of ranking terms by probability.

Comparison with other methods. To validate the developed OrthoML2GO method, it was compared with the Blast2GO (Conesa et al., 2005) and PANNZER2 (Törönen et al., 2018) methods. BLAST homology searches were launched on the computational complex of the “Bioinformatics” collective use center at ICG SB RAS. The launch parameters for Blast2GO and PANNZER2 were run with default parameters.

## Results and discussion

**Impact of orthogroup information on GO term prediction performance**

To assess the influence of orthogroup information on function prediction performance, a comparison of the F1-score was conducted for three annotation methods with three algorithms (KNN, OG, and KNN+OG) depending on the number of nearest homologs for A. thaliana sequences (Fig. 2).

As shown in Figure 2, the F1-score depends on the parameter k for all three annotation variants. However, the nature of these dependencies is different: OG demonstrates the lowest performance (F1 < 41 %). For the OG method, as for the other methods, a maximum is observed at k = 15. Moreover, increasing the parameter k results in a gradual, albeit slight, decrease in the F1-score. For the most accurate prediction, determining the correct orthologous group of the protein, which can be identified even at small values of k, is sufficient. A further increase in k only adds noise to the prediction due to an increase in false positive GO terms from orthogroups to which the protein in question does not actually belong.

The KNN method shows a pronounced dependence of performance on the parameter k. At small values (k = 5), the F1-score is the lowest (~40 %) and lower than the OG and KNN+OG methods, which is probably due to an insufficient number of homologs for reliable statistical inference and high sensitivity to noise and potential annotation errors of individual sequences. When k increases to 15, F1 grows to a maximum value (~52 %); however, a further increase in k leads to a gradual decrease in performance, as distant homologs which may carry functionally irrelevant information for the target sequence (false positive GO terms) begin to enter the sample.

Note that combining the KNN and OG methods (KNN+OG) leads to an increase in the F1-score for all values of the parameter k, and the greatest increase (more than 3 % in absolute value) is observed precisely at k = 5. This can be explained by the fact that with small k, the list of homologs may be unstable and statistically unreliable. Incorporating orthogroup information, which aggregates data on the function of a whole group of evolutionarily related genes, stabilizes the prediction and compensates for the insufficiency of data from a small number of nearest neighbors.

It is worth noting that the F1-score value in the range of 40–52 % represents a competitive result for the task of protein function prediction, as confirmed by comparison with other popular methods (see section “Comparison of the performance of KNN, KNN+OG, and OrthoML2GO with the Blast2GO and PANNZER2 tools”). This is due to the rather complex nature of the task: firstly, as mentioned earlier, GO annotation is multiple, i. e., one protein corresponds to many terms, and the prediction is considered correct only if all correct terms are found and no extra ones are added. Secondly, the distribution of GO terms is extremely imbalanced: some terms are very common, others are extremely rare, which further complicates achieving high accuracy. Thus, the absolute value of the F1-score should be interpreted in the context of the task’s complexity and in comparison, to alternative approaches.

Results for other organisms are shown in the Supplementary materials (Tables S4–S9). Combining the KNN and OG methods (KNN+OG) allows us to obtain an integrated prediction that demonstrates the greatest gain in accuracy at small values of the parameter k for all organisms except Chlamydomonas reinhardtii. For example, for Danio rerio proteins at k = 5, the KNN+OG method surpasses the basic KNN by more than 13 % in absolute value of the F1-score (74.66 vs. 61.37 %). This is explained by the fact that with small k, the list of homologs may be statistically unreliable and sensitive to noise in the annotations of individual sequences. Integrating orthogroup data mitigates the statistical unreliability associated with a small number of nearest homologs. Thus, the hybrid KNN+OG approach not only demonstrates the best performance at the peak (at k = 15) but also significantly reduces the dependence of prediction accuracy on the parameter k, making the method more robust.

Thus, combining the KNN and OG variants (KNN+OG) allows obtaining an integrated prediction, giving a better estimate compared to each of them individually for all values of the parameter k for most organisms, and it will be used for machine learning

**Verification of GO terms by various machine learning algorithms**

To verify false positive GO terms obtained at the previous stage, machine learning algorithms such as logistic regression (LR), gradient boosting (XGB), and random forest (RF) were used (see section “Verification of terms using machine learning methods”). A comparison of the accuracy of machine learning methods using the Fmax measure (see section “Evaluation metrics”) on test data of A. thaliana, H. sapiens, and a combined sample of 20,000 sequences from different organisms is presented in Table 2.

Logistic regression demonstrates significantly lower Fmax values compared to gradient boosting and random forest methods, with the difference reaching over 25 %. This is likely due to the fact that ensemble methods (XGB and RF), unlike the linear LR model, are capable of capturing complex nonlinear relationships between features. Furthermore, these methods are more robust to noise in the data due to bagging (RF) and boosting (XGB) procedures, which average the predictions of many individual decision trees, reducing the influence of outliers and incorrect annotations of individual proteins. Gradient boosting (XGB) demonstrates the best results on Arabidopsis sequences and the general sample of all organisms, but it only slightly trails the random forest method on human proteins (with an Fmax difference of only 0.1 %). Thus, for the final version of the OrthoML2GO method, the gradient boosting (XGB) machine learning method was chosen, as it showed the best results on the test samples.

**Comparison of the performance of KNN, KNN+OG, and OrthoML2GO with the Blast2GO and PANNZER2 tools**

For a comprehensive assessment of the developed method’s effectiveness, its performance was compared with two widely used automatic functional annotation tools – Blast2GO and PANNZER2. The comparison was performed on three test datasets: individual proteomes of A. thaliana and H. sapiens, as well as a combined sample including sequences of all organisms listed in Table 1. As the resulting metric for methods not using machine learning (KNN, KNN+OG, Blast2GO), the F1- score was applied, while for OrthoML2GO and PANNZER2, which output a probabilistic estimate, the Fmax metric was used, allowing us to evaluate the maximum achievable quality of the model with an ideal choice of cutoff threshold (Table 3).

Analysis of the results demonstrates that the developed OrthoML2GO method, integrating homology search, orthogroup analysis, and verification of GO terms using gradient boosting, shows a statistically significant advantage in performance over all compared methods on all test samples. Thus, for A. thaliana, OrthoML2GO achieved an Fmax of 68.95 %. This represents an 18.21 % increase over PANNZER2 (Fmax = 50.74 %) and a 14.65 % increase over the F1-score of Blast2GO (54.30 %). On human proteins, compared to PANNZER2, OrthoML2GO performed significantly better – 83.92 vs. 75.14 %, while for the Blast2GO method, the F1 value was 54.95 %. On the combined sample of all organisms, an improvement in the F-measure indicator of more than 30 % was observed compared to all other methods.

Notably, the hybrid KNN+OG approach, which underlies OrthoML2GO, demonstrates a small but consistent improvement compared to the basic KNN on all samples, confirming the usefulness of integrating orthogroup information. However, the main gain in accuracy is provided by gradient boosting (XGB), which effectively verifies false positive predictions arising from annotation noise.

A key factor contributing to the success of the OrthoML2GO method is its integration of evolutionary information from homologs and orthogroups within the OrthoDB database, combined with subsequent verification of GO terms using gradient boosting. In contrast to PANNZER2 and Blast2GO, our method incorporates orthogroup information and verifies GO terms using decision tree ensembles, adaptively selecting the most informative features. Ultimately, this allowed reducing the proportion of false positive annotations and increasing accuracy from 8 % (on human protein sequences) to 30 % (on the combined sample) compared to analogues.

It is important to note a potential limitation in the comparison: our machine learning models were trained on a sample of sequences from OrthoDB, while Blast2GO and PANNZER2 rely on broader datasets derived from UniProt. This difference in training data may introduce a bias in the comparative accuracy estimates

**Assessment of prediction performance for different GO aspects**

For a more detailed analysis of the method’s performance, a comparative analysis of the prediction accuracy of GO terms for the three main aspects (ontologies) of Gene Ontology was performed: Biological Process (BP), Molecular Function (MF), and Cellular Component (CC). The evaluation results on the combined sample for various machine learning algorithms used at the verification stage are presented in Table 4.

The results show that all machine learning algorithms demonstrate a similar trend: the highest prediction accuracy is achieved for the Cellular Component (CC) aspect, followed by Molecular Function (MF), and the accuracy is somewhat lower for Biological Process (BP). This is consistent with the generally accepted view in bioinformatics: predicting cellular localization (CC) is often the easiest task, as it strongly correlates with the presence of specific signal peptides and domains. Prediction of molecular function (MF) also largely depends on conserved functional domains. At the same time, prediction of involvement in biological processes (BP) is the most complex, as the same protein can participate in several processes, and the processes themselves are defined by complex interactions of many proteins, which is more difficult to deduce solely from homology and orthology data

The XGB method, chosen for OrthoML2GO, demonstrated the best results among all tested algorithms across all three aspects, further confirming its suitability as the final classifier. The performance of our method is competitive with the accuracy estimates of other methods reported in the literature (Table 5). The comparison was performed using the Fmax metric for individual Gene Ontology aspects: BP – biological processes, MF – molecular functions, CC – cellular components.

It can be noted that the OrthoML2GO method (Table 4) demonstrated competitive results: 78.8 % (BP), 79.8 % (MF), and 83.6 % (CC) on a sample of 20,000 sequences from seven heterogeneous organisms – both plants and animals. Upon comparison, it is evident that OrthoML2GO surpasses most of the studied methods in all aspects. However, PANNZER2 showed higher values for MF (85.8 %) and CC (85.3 %), albeit on a smaller and less diverse sample (5,000 sequences from Swiss-Prot).

It is worth noting that direct quantitative comparison with other methods may be complicated by methodological differences. Firstly, test samples differ significantly: most methods use the UniProt/Swiss-Prot database, while our combined sample includes both plants and animals, which may affect the comparability of results. Secondly, the version of Gene Ontology is critically important: OrthoML2GO relies on the latest version of OrthoDB v12 annotation (GO 2025), which may lead to difficulties in comparing quality metrics.

To demonstrate the applicability of the OrthoML2GO method to poorly studied organisms, the proteome of the green alga Ostreococcus lucimarinus was annotated (Tables S10 and S11). The method predicted functions for 5,273 out of 7,603 protein sequences. The analysis revealed a predominance of such biological processes as phosphorylation (GO:0016310) and translation (GO:0006412). Among molecular functions, ATP binding (GO:0005524) and nucleotide binding (GO:0000166) were the most frequent, and among cellular components, membrane (GO:0016020) and nucleus (GO:0005634). These results demonstrate the method’s ability to annotate poorly studied proteomes and identify functional profiles characteristic of non-model organisms.

## Conclusion

The developed method, OrthoML2GO, which integrates homology searches and orthogroup analysis from the OrthoDB database with gradient boosting, demonstrated high efficiency on test samples. One of the main results is a significant improvement in annotation accuracy due to the combined approach, which combines the k-nearest neighbors method and information about orthologous groups (KNN+OG). This hybrid method surpassed the individual KNN and OG approaches, especially at small values of the parameter k. Verification of GO terms using machine learning algorithms, particularly gradient boosting (XGB), allowed for a further increase in accuracy through effective filtering of false positive predictions arising from distant homologs and orthogroups.

The obtained results confirm that the use of evolutionary information contained in the OrthoDB orthogroups, combined with machine learning algorithms, is an effective strategy for automatic prediction of protein sequence functions. The proposed OrthoML2GO method can be a good alternative to existing methods. It is worth noting that further improvement in accuracy is possible by optimizing machine learning parameters, as well as by including additional sources of biological information. As prospects for further research, the following directions are outlined: evaluation of the model’s transferability to poorly annotated proteomes and comparative analysis with other methods using machine learning, including neural network-based onees.

## Conflict of interest

The authors declare no conflict of interest.
